# Re‐appraisal of the Diagnostic Value of PAS‐D Staining in TTF‐1‐NEGATIVE NSCLC Subclassification: A Large Real‐World Cohort Study

**DOI:** 10.1111/pin.70154

**Published:** 2026-07-26

**Authors:** Dogu Vuralli Bakkaloglu, Hafize Ozdemir, Melek Buyuk, Dilek Yilmazbayhan, Yasemin Ozluk

**Affiliations:** ^1^ Department of Pathology, Istanbul Faculty of Medicine Istanbul University Istanbul Türkiye

**Keywords:** cytology, mucin stain, non‐small cell lung carcinoma (NSCLC), NSCLC subclassification, PAS‐D, poorly differentiated adenocarcinoma, TTF‐1‐negative adenocarcinoma (TTF‐1neg ADC)

## Abstract

Subclassification of NSCLC is critical for therapeutic decision‐making, particularly in small biopsies and cytology specimens where ancillary testing must be limited. Although the TTF‐1/p40 panel is widely used to distinguish adenocarcinoma (ADC) from squamous cell carcinoma, a subset of NSCLCs remains unclassified, highlighting the need for adjunctive markers. Periodic acid‐Schiff with diastase (PAS‐D) is readily available but underutilized. We retrospectively reviewed 2611 NSCLC specimens from 2298 patients. Diagnosis was based on morphology alone in 38.6% of cases, while ancillary testing was required in 57.7%. Among 287 TTF‐1_neg_/p40_neg_ tumors, 30.3% were classified as ADC using PAS‐D and/or Napsin A. PAS‐D demonstrated higher sensitivity than Napsin A (71.8% vs. 69.3%), and markedly outperformed Napsin A in TTF‐1‐negative ADCs (89.7% vs. 25.9%, *p* < 0.001). This finding reflects biological differences between terminal respiratory unit‐derived ADCs and non‐terminal airway tumors, in which PAS‐D positivity is more diagnostically informative. Combined testing improved sensitivity, with the TTF‐1/PAS‐D panel achieving 97.5%. This study highlights the underappreciated diagnostic value of PAS‐D, particularly in TTF‐1‐negative tumors. PAS‐D represents a simple, effective adjunct to routine diagnostic panels. Therefore, incorporating PAS‐D into routine panels can enhance NSCLC subclassification, particularly in TTF‐1_neg_/p40_neg_ tumors and support accurate diagnosis and optimal clinical management.

## Graphical abstract

- Combining TTF‐1 with PAS‐D increased sensitivity for ADC to 97.5%, whereas TTF‐1 with Napsin A reached only 84.3%.
- PAS‐D alone demonstrated 89.7% sensitivity for TTF‐1‐negative ADCs, while Napsin A achieved only 25.9%.

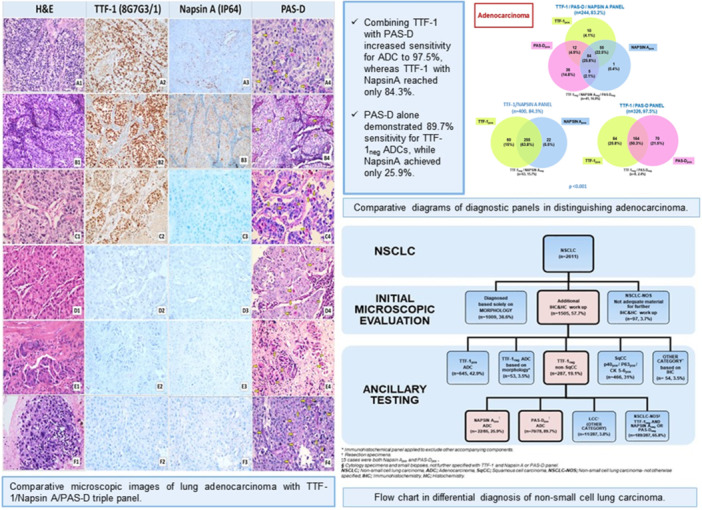

## 1 Introduction

Histopathological subclassification of non‐small cell lung carcinoma (NSCLC) has gained increasing importance with the advent of targeted therapies that directly influence treatment decisions and patient outcomes [1, 2]. Given that most NSCLC cases present at advanced stages, diagnosis often relies on small biopsies and cytological specimens. Morphological features such as keratin pearls for squamous cell carcinoma (SqCC) and glandular structures with mucin droplets for adenocarcinoma (ADC), can often suffice for diagnosis. When needed, concise ancillary panels are crucial to preserve material for molecular studies while achieving accurate subtyping.

The immunohistochemical panel of thyroid transcription factor 1 (TTF‐1) and p40 has been widely accepted for routine subclassification based on their high specificity and mutual exclusivity [1, 3, 4, 5]. However, a persistent challenge remains: a significant subset of NSCLC cases that are negative for both TTF‐1 and p40 cannot be reliably classified using this panel alone. These cases, often designated as NSCLC‐ not otherwise specified (NSCLC‐NOS), are typically managed as adenocarcinoma in clinical settings if they are negative for squamous markers, yet they remain a diagnostic gray zone.

While mucin stains such as PAS‐D have historically aided in identifying glandular differentiation, their routine use has diminished in the era of immunohistochemistry and molecular diagnostics. Nonetheless, PAS‐D remains accessible, cost‐effective, and technically simple‐ features that make it particularly relevant in settings where sample size or resources are limited. Despite this, there is limited real‐world data evaluating PAS‐D's diagnostic contribution, especially in cytological materials and TTF‐1−negative NSCLC cases.

To address this gap, we conducted a comprehensive retrospective study of over 2600 lung specimens diagnosed at a single tertiary center over 10 years. Our primary aim was to assess the practical diagnostic value of PAS‐D in NSCLC subclassification‐ particularly TTF‐1−negative cases and to compare its utility to that of Napsin A. In doing so, we also sought to reflect a decade of routine practice with emphasis on real‐life applicability and the evolving diagnostic landscape.

## 2 Material and Methods

### 2.1 Study Design

A total of 3278 lung specimens diagnosed as carcinoma at Istanbul University, Istanbul Faculty of Medicine, Department of Pathology, between 2011 and 2020 were retrospectively reviewed. Neuroendocrine, salivary gland‐type, and metastatic tumors were excluded. To ensure accurate assignment of tumor origin, cases with uncertain or incompletely documented primary tumor status were systematically re‐evaluated using the institutional electronic medical record system. In diagnostically challenging cases, radiologic findings, including computed tomography (CT) and positron emission tomography (PET) imaging, as well as multidisciplinary tumor board reports, were reviewed. Only tumors with sufficient clinicopathologic evidence supporting a primary pulmonary origin were included in the final study cohort, whereas metastatic tumors and cases with unresolved primary site assignment were excluded.

The final study cohort included 2611 lung cytology, small biopsy, and resection specimens from 2298 patients. Cytological specimens included formalin‐fixed paraffin‐embedded (FFPE) cell blocks from transthoracic and transbronchial needle aspirations (TTNA, TBNA), bronchial brushings, lavages, sputum samples and pleural effusions.

Demographic data, histopathological diagnoses, and additional IHC and histochemical work‐ups were extracted from pathology reports. All cases had originally been evaluated at the time of diagnosis by one of two experienced thoracic pathologists/cytopathologists (YO and DY) working within a unified institutional diagnostic framework and applying consistent diagnostic criteria throughout the study period. During data retrieval, cases with uncertain subclassification or unresolved clinicopathologic questions underwent targeted re‐review by two thoracic pathologists/cytopathologists involved in the study (DVB and YO).

Between 2011 and 2020, our diagnostic protocols evolved in line with WHO [3, 6] and IASLC [7] recommendations. After 2011, routine application of TTF‐1 (clone 8G7G3/1) and p40 (clone BC 28) became standard in all NSCLC diagnoses. Napsin A (clone IP64) was gradually adopted from 2014 onwards, primarily in TTF‐1‐negative cases.

Depending on reagent availability over the years, additional squamous markers‐ including p63 (clone 4A4), and CK5/6 (clone D5/16B4) were applied in selected cases when deemed necessary for accurate subclassification. Due to the retrospective nature of the study and evolving diagnostic practices over the years, ancillary stains were not applied uniformly across all cases. However, all NSCLC‐NOS and LCC cases underwent at least TTF‐1 and p40 immunohistochemistry. In cases negative for both markers, PAS‐D and/or Napsin A were added when sufficient tissue was available. A more standardized diagnostic approach was consistently adopted in later years.

Clinical, radiological and pathological correlation was used to exclude metastasis. For TTF‐1‐negative tumors with atypical morphology, further IHC workup (CK7, CK20, GATA3, etc.) was applied if clinically indicated.

The diagnostic criteria for subclassification were based on WHO/IASLC guidelines [3, 6, 7] and defined as follows: Cases showing positivity for pneumocyte markers (TTF‐1 and/or Napsin A) and/or mucin stains, in the absence of squamous marker expression (p40 and/or p63), were classified as ADC. Conversely, cases positive for squamous markers and negative for pneumocyte markers and/or mucin stains were classified as SqCC. Positivity thresholds were as follows: ≥ 10% tumor cell staining for TTF‐1 and Napsin A, and ≥ 50% for p40, and CK5/6. Additionally, tumors exhibiting focal expression of TTF‐1 and/or Napsin A in even a small subset of tumor cells, in the absence or minimal presence of squamous marker expression (excluding entrapped alveolar epithelium), were also classified as ADC.

PAS‐D staining was performed according to the routine laboratory protocols implemented during the study period. Depending on the period of diagnosis, staining was carried out either manually or using automated staining platforms with standardized commercial reagents following the manufacturers' validated protocols. In all cases, tissue sections underwent enzymatic glycogen digestion with diastase (α‐amylase) prior to the periodic acid‐Schiff reaction to enhance the specificity of intracellular mucin detection. Intracellular mucin positivity was defined as mucin droplets present in at least five tumor cells across two high‐power fields, confirmed by PAS‐D staining [3, 6]. PAS‐D positivity was defined by the presence of intracytoplasmic mucin droplets exhibiting a soap‐bubble, foamy, or targetoid appearance.

Most of the cases included in this study were diagnosed after the publication of the 2011 IASLC/ATS/ERS classification for lung ADC, which recommends that tumors expressing both TTF‐1 and any squamous marker be classified as NSCLC, favor ADC [7]. To ensure consistency with current standards, all cases initially diagnosed as LCC (*n* = 32) were retrospectively re‐evaluated based on the 2021 WHO classification. Only resection specimens that lacked glandular or squamous differentiation by both morphology and ancillary testing (IHC/HC) were retained as true LCC. For these cases, nuclear markers (TTF‐1 and p40) and cytoplasmic markers (Pancytokeratin, Napsin A and cytokeratin 5/6) were applied in a complementary manner to minimize technical artifacts and enhance diagnostic reliability. PAS‐D staining was performed in all re‐evaluated cases. All IHC and PAS‐D stains were applied to two paraffin blocks selected to include areas most likely to exhibit morphologic differentiation. Neuroendocrine markers (chromogranin, synaptophysin, CD56, INSM1) were added when neuroendocrine morphology was suspected.

Molecular testing was not systematically performed in this study. Most cases consisted of small biopsies or cytology specimens with limited tumor content, which hindered DNA‐based analyzes. Additionally, the retrospective nature of the study and lack of research funding meant molecular studies were not part of the original protocol. Only a small subset of cases had molecular results, and the tests performed were not standardized. Furthermore, the samples had been archived for over 5 years, limiting their suitability for reliable molecular analysis.

### 2.2 Statistical Analysis

Data were analyzed using the IBM Statistical Package for Social Sciences software system (SPSS version 28). Descriptive statistics are reported as mean±standard deviation for continuous variables, and frequency and percentages for categorical variables. Categorical variables were compared using Pearson's chi‐square or Fisher's exact tests as appropriate. A *p*‐value of < 0.05 was considered statistically significant.

### 2.3 Ethical Approval

This study was approved by the Clinical Research Ethics Committee of Istanbul University, Istanbul Faculty of Medicine (2024‐176).

## 3 Results

### 3.1 Demographic Data

Among 2298 patients, 80.8% were male. The mean age was 63.2 ± 9.4 (range: 21–95) for males and 60.8 ± 10.9 (range: 14–90) for females. Following the re‐evaluation of cases initially diagnosed as LCC, the revised histological subtype distribution was as follows: ADC, 1028 (44.7%); SqCC, 954 (41.5%); NSCLC‐NOS, 255 (11.1%), and other less frequent subtypes, 61 (2.7%; 11 LCC, 22 ASC, 26 SC, one lymphoepithelial carcinoma (LEC) and one NUT carcinoma). SqCC was the most common histologic subtype in men (46%), whereas ADC predominated in women (64.9%).

### 3.2 Sampling Methods

Cytological specimens were predominant (38.3%), followed by small biopsies (35.2%) and resections (26.5%). ADCs were primarily diagnosed by cytology (43.3%), especially transthoracic fine needle aspirations (FNAs), consistent with their peripheral location. SqCCs (49.1%) were mostly diagnosed via small biopsies, notably bronchoscopic biopsies, reflecting their central localization. Sampling methods and corresponding histological subtype distributions are detailed in Table 1.

**Table 1:** The distribution of histological subtypes according to sampling methods.

All specimens (*n* = 2611)	ADC (*n* = 1159)	SqCC (*n* = 1101)	NSCLC‐NOS (*n* = 286)	Other (*n* = 65)
Cytology* (*n* = 1000, 38.3%)	502 (43.3%)	283 (25.7%)	200 (69.9%)	15 (23.1%)
Small biopsies† (*n* = 918, 35.2%)	286 (24.7%)	541 (49.1%)	81 (28.3%)	10 (15.4%)
Resections‡ (*n* = 693, 26.5%)	371 (32.0%)	277 (25.2%)	5^§^ (1.8%)	40 (61.5%)

*Note:* Other category includes large cell carcinoma, adenosquamous carcinoma, sarcomatoid carcinoma, lymphoepithelial carcinoma and NUT carcinoma.

Abbreviations: ADC, adenocarcinoma; NSCLC‐NOS, non‐small cell lung carcinoma–not otherwise specified; SqCC, squamous cell carcinoma.

* Sputum, bronchial lavage, bronchoalveolar lavage, bronchial brushing, transbronchial fine needle aspiration, transthoracic fine needle aspiration.

^†^ Bronchoscopic biopsy, transthoracic trucut biopsy.

^‡^ Wedge resection, segmentectomy, trisegmentectomy, lobectomy, bilobectomy, pneumonectomy.

^§^ Five patients who were initially diagnosed as non‐ small cell carcinoma had major pathologic response after neoadjuvant chemoradiotherapy.

### 3.3 Subclassification of NSCLC

Ninety‐seven specimens (3.7%); including 84 cytological samples, 11 small biopsies, and 2 resections (due to major pathologic response following neoadjuvant chemoradiotherapy), were inadequate for ancillary testing. Among the remaining 2514 specimens, 1009 (38.6%) exhibiting clear squamous and/or glandular features were diagnosed based solely on morphology, whereas 1505 (57.7%) required additional IHC & histochemical (HC) analyzes. The detailed necessity for further testing by histologic subtype and sampling method is shown in Table 2.

**Table 2:** Necessity of additional immunohistochemical and histochemical work‐up according to histomorphological subtypes and sampling methods (*n* = 2514).

	Diagnosed based on morphology (*n* = 1009)	Diagnosed based on additional IHC and HC (*n* = 1505)	Total (*n* = 2514)*
Cytology (*n* = 916)			
ADC	168 (33.5%)	334 (66.5%)	502 (100%)
SqCC	153 (54.1%)	130 (46%)	283 (100%)
NSCLC‐NOS	*	116 (100%)	116 (100%)
Other^‡^	—	15 (100%)	15 (100%)
Total	321 (35%)	595 (65%)	916 (100%)
Small biopsy (*n* = 907)			
ADC	50 (17.5%)	236 (82.5%)	286 (100%)
SqCC	292 (54%)	249 (46%)	541 (100%)
NSCLC‐NOS	*	70 (100%)	70 (100%)
Other^‡^	—	10 (100%)	10 (100%)
Total	342 (37.7%)	565 (62.3%)	907 (100%)
RESECTION (*n* = 691)			
ADC	156 (42%)	215 (58%)	371 (100%)
SqCC	190 (68.6%)	87 (31.4%)	277 (100%)
NSCLC‐NOS	*	3 (100%)†	3 (100%)†
Other‡	—	40 (100%)	40 (100%)
Total	346 (50.1%)	345 (49.9%)	691 (100%)

Abbreviations: ADC, adenocarcinoma; HC, histochemistry; IHC, immunohistochemistry; NSCLC‐NOS, non‐small cell lung carcinoma, not otherwise specified; SqCC, squamous cell carcinoma.

* Specimens (*n* = 97) that are not sufficient for further diagnostic tests are not included in the total count.

^†^ Resections with major pathologic response after neoadjuvant chemoradiotherapy.

^‡^ Other category includes large cell carcinoma, adenosquamous cell carcinoma, sarcomatoid carcinoma, lymphoepithelial carcinoma and NUT carcinoma.

**Figure 1:**
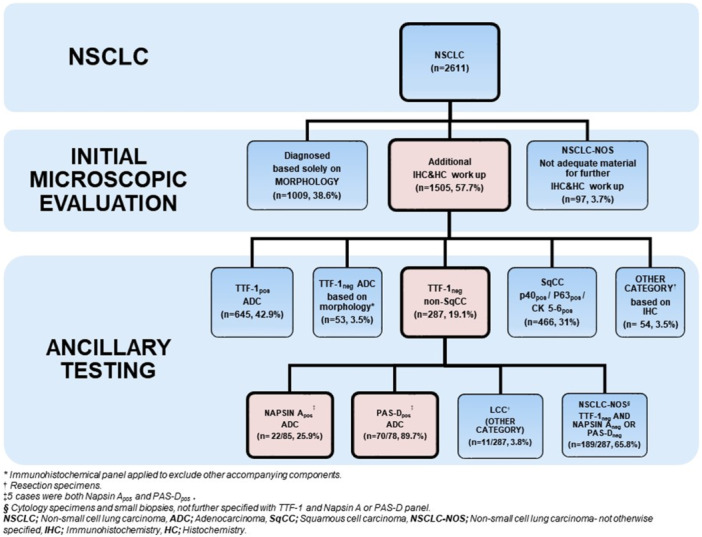
Flow chart in differential diagnosis of non‐small cell lung carcinoma.

Ancillary testing identified 287 cases (19.1%) as TTF‐1‐neg/p40‐neg. Within this group, 87 (30.3%) were classified as ADC using PAS‐D and/or Napsin A in addition to TTF‐1 and p40. The ancillary testing flowchart is shown in Figure 1.

### 3.4 Detailed Histomorphological Findings of Cases Previously Diagnosed as Large Cell Carcinoma

Of the 32 LCC cases, 21 were reclassified and 11 remained in LCC. Fourteen (43.8%) were reclassified as solid ADC; TTF‐1 was focally positive in 6 (42.9%), Napsin A was focally positive in 3 (21.4%; all TTF‐1 positive), and PAS‐D was positive in 11 (78.6%), with 8 (57.1%) diagnosed solely on PAS‐D positivity. Five cases (15.6%) were reclassified as poorly differentiated SqCC, positive for p40 and CK5/6. One case (3.1%) previously diagnosed as LEC was reclassified under “other” due to Epstein‐Barr virus‐encoded small RNA (EBER) positivity and diffuse p40 staining. Another (3.1%) was pleomorphic carcinoma with mixed SqCC and giant cell components. No neuroendocrine carcinomas were identified.

Re‐defined ADC cases displayed rhabdoid features (3, 21.4%), clear cell morphology (2, 14.3%) or no significant differentiation (9, 64.3%). SqCC cases showed basaloid features (3, 60%), clear cell morphology (1, 20%), or no obvious differentiation (1, 20%). PAS‐D (78.6%) outperformed both TTF‐1 (42.9%) and Napsin A (21.4%) in identifying poorly differentiated solid ADC (*p* < 0.001).

### 3.5 Comparison of Diagnostic Markers for Adenocarcinoma

Among 1159 ADC specimens, 458 (39.5%) were diagnosed based on glandular morphology alone with hematoxylin and eosin (H&E) staining. Eighty‐four (7.2%) showed uncertain components or required confirmation of primary tumor status, necessitating further investigation. The remaining 701 (60.5%) specimens lacked clear morphological features and underwent brief IHC/HC panels including at least one pneumocyte marker (TTF‐1/Napsin A/PAS‐D) and one squamous marker (p40/CK5‐6/p63).

Of these, 645 cases (82.2%) were TTF‐1 positive. Among 140 TTF‐1‐neg ADCs, 70 (50%) were PAS‐D positive and 22 (15.7%) Napsin A positive, and 5 (3.6%) demonstrated concurrent positivity. Fifty‐three (37.9%) TTF‐1_neg_ cases exhibited glandular morphology without immunohistochemical evidence of other components. The diagnostic sensitivities of the individual ADC markers varied across different specimen types. For TTF‐1, the sensitivity was 81.7% (273/334) in cytology, 82.2% (194/236) in small biopsies, and 82.8% (178/215) in resections, yielding an overall sensitivity of 82.2% (*n* = 785). PAS‐D exhibited sensitivities of 62.1% (64/103) in cytology, 73.5% (72/98) in small biopsies, and 78.4% (98/125) in resections, with an overall sensitivity of 71.8% (*n* = 326). Lastly, Napsin A demonstrated sensitivities of 74.7% (136/182) in cytology, 58.6% (58/99) in small biopsies, and 69.7% (83/119) in resections, resulting in an overall sensitivity of 69.3% (*n* = 400).

**Figure 2:**
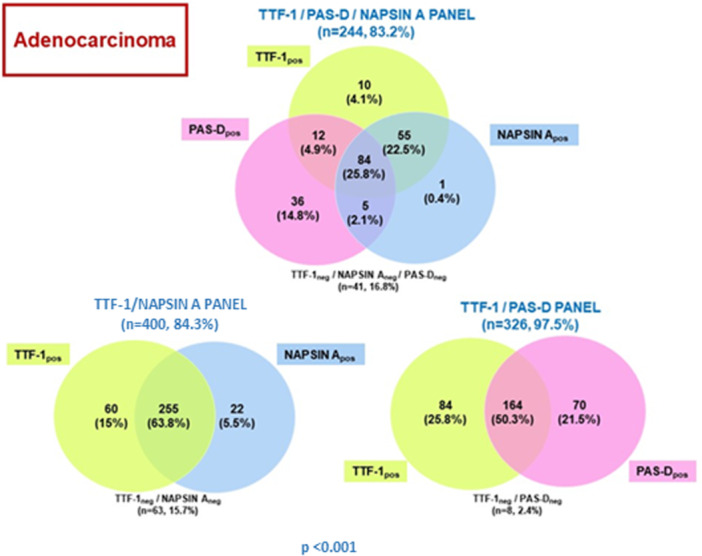
Diagrams of diagnostic panels in distinguishing adenocarcinoma (data gathered from cases where multiple panels were available).

**Figure 3:**
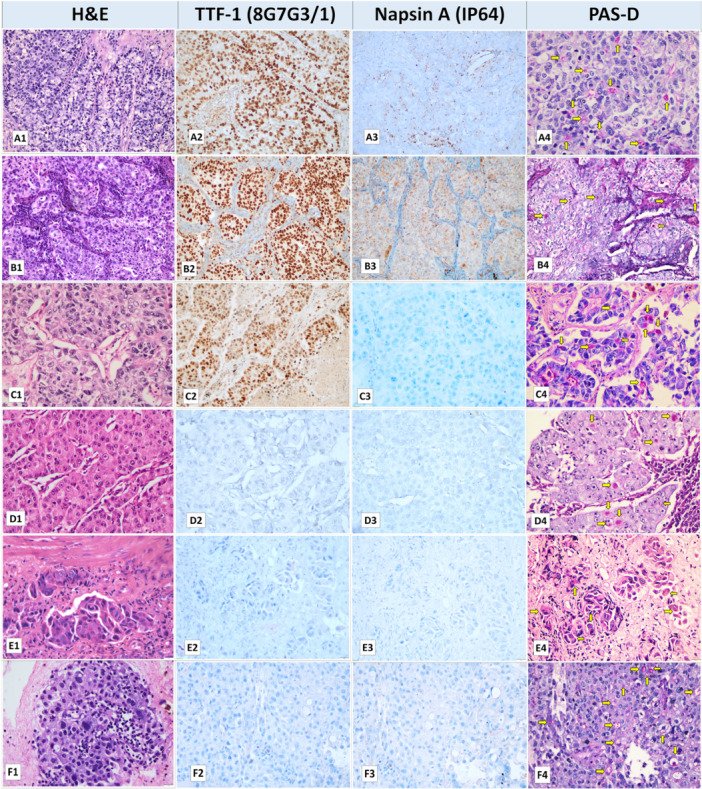
Expressions of TTF‐1, NapsinA and PAS‐D in clinically confirmed primary lung adenocarcinomas. A1‐4 and B1‐4 (Resection, x400): TTF‐1_pos_/NapsinA_pos_/PAS‐D_pos_ adenocarcinoma. C1‐4 (Resection, x400): TTF‐1_pos_/NapsinA_neg_/PAS‐D_pos_ adenocarcinoma. D1‐4 (Resection, x400), E1‐4 (Bronchoscopic biopsy, x400) and F1‐4 (Cell block of transthoracic fine needle aspiration, x400): TTF‐1_neg_/NapsinA_neg_/PAS‐D_pos_ adenocarcinoma. A4‐F4: Arrows highlighting PAS‐D_pos_ intracytoplasmic mucin droplets (x400).

Double or triple IHC/HC panels increased sensitivity: TTF‐1/Napsin A to 84.3% (*n* = 400), TTF‐1/PAS‐D to 97.5% (*n* = 326), and TTF‐1/PAS‐D/Napsin A to 83.2% (*n* = 244). Schematic diagrams of these diagnostic co‐test panels, microscopic images of ADC cases evaluated with triple IHC/HC panel, and co‐test results for ADCs diagnosed via cytological specimens are provided in Figures 2 and 3 and Table 3, respectively.

**Table 3:** The results of ancillary tests in the evaluation of adenocarcinoma cases diagnosed by cytological specimens.

Adenocarcinoma cases diagnosed by cytological specimens				
	TTF‐1 AND PAS‐D CO‐TEST (*n* = 144)		TTF‐1 AND NAPSIN A CO‐TEST (*n* = 182)	
	PAS‐D_pos_	PAS‐D_neg_	NAPSIN A_pos_	NAPSIN A_neg_
TTF‐1_pos_	45 (31.2%)*	35 (24.3%)	121 (66.5%)*	22 (12.1%)
TTF‐1_neg_	21 (14.6%)†	43 (29.9%)	15 (8.2%)†	24 (13.2%)
Total	66 (45.8%)	78 (54.2%)	136 (74.7%)	46 (25.3%)

* 26 cases were TTF‐1_pos_/PAS‐D_pos_/Napsin A_pos_.

^†^ 3 cases were TTF‐1neg/PAS‐Dpos/Napsin Apos.

Among the TTF‐1‐negative cases, PAS‐D confirmed ADC in 89.7% (70/78) of cases using the TTF‐1/PAS‐D panel, whereas Napsin A identified only 25.9% (*n* = 22/85) via the TTF‐1/Napsin A panel (89.7% vs 25.9%; *p* < 0.001). The distribution of PAS‐D and Napsin A positivity according to specimen type in TTF‐1‐negative ADCs is outlined in Table 4.

**Table 4:** The distribution of PAS‐D and Napsin A positivity according to specimen type in TTF‐1 negative adenocarcinomas.

		PAS‐D (*n* = 78)		NAPSIN A (*n* = 85)	
		PAS‐D_pos_ (*n* = 70)	PAS‐D_neg_ (*n* = 8)	NAPSIN A_pos_ (*n* = 22)	NAPSIN A_neg_ (*n* = 63)
Cytology	Transbronchoscopic brushing Transbronchial FNA Bronchial lavage	4 (100%)	0 (0%)	1 (25%)	4 (75%)
	Transthoracic FNA*	16 (76.2%)	5 (23.8%)	14 (40%)	21 (60%)
Small biopsy	Bronchial biopsy	18 (94.7%)	1 (5.3%)	0 (0%)	16 (100%)
	Transthoracic trucut biopsy	8 (88.9%)	1 (11.1%)	0 (0%)	5 (100%)
Resection		24 (96%)	1 (4%)	7 (28%)	18 (72%)

* FNA: Fine needle aspiration.

Forty‐six ADC cases (5.9%) showed focal p63 positivity (≤ 20%), of which 45 (97.8%) were TTF‐1−positive and one PAS‐D−positive. Only three ADC cases exhibited focal p40 and CK5/6 positivity (≤ 10%), all concurrent with TTF‐1 positivity. No significant co‐expression of pneumocyte and squamous markers complicated NSCLC subclassification.

## 4 Discussion

In this large‐scale single‐center retrospective study, we reviewed 2611 lung specimens collected over a decade to assess the real‐world performance of routine ancillary tests for NSCLC subclassification. Our findings confirm the long‐standing value of TTF‐1 as the most sensitive marker (82.2%) for ADC, PAS‐D achieved a sensitivity of 71.8% and demonstrated a markedly higher diagnostic yield than Napsin A in TTF‐1‐negative ADCs (89.7% vs. 25.9%), highlighting its potential role in resolving a persistent diagnostic gap in routine pulmonary practice.

Mucin staining has long been recognized as a reliable indicator of glandular differentiation, although its use has progressively declined with advances in immunohistochemistry and molecular diagnostics. PAS‐D provides a broad assessment of intracellular mucosubstances by combining enzymatic glycogen digestion prior to the periodic acid‐Schiff reaction, thereby facilitating the detection of intracellular secretory material while minimizing glycogen‐related interpretative pitfalls. This is especially relevant in poorly differentiated pulmonary ADCs, where mucin production may be limited to minute intracellular droplets and mucin profiles are often heterogeneous. Compared with mucicarmine, which is relatively specific for epithelial acidic mucins, PAS‐D has a broader staining spectrum and may better detect focal intracellular mucin production [8]. Earlier studies demonstrated the superior sensitivity of PAS‐D over other mucin stains, supporting its role as a comprehensive histochemical approach for demonstrating glandular differentiation in diagnostically challenging NSCLC cases [9, 10, 11].

Although mucin staining has been recommended in international guidelines for more than a decade, contemporary systematic studies evaluating its diagnostic performance across different specimen types remain scarce. One study evaluating bronchial biopsies and paired resections demonstrated substantial diagnostic shifts after incorporating AB‐PAS staining and concluded that the TTF‐1/p63/AB‐PAS panel was optimal for distinguishing ADC and SqCC. Similar to our findings, the addition of mucin staining improved the sensitivity of ADC diagnosis (69% vs 54%) [12]. In our cohort, TTF‐1/PAS‐D co‐test achieved the highest sensitivity 97.5% among all evaluated marker combinations.

Another study comparing PAS‐D, AB‐PAS and mucicarmine demonstrated the superior sensitivity of PAS‐D for pulmonary ADC (51% vs. 48% and 31%, respectively) [8]. Similar to our results, PAS‐D and AB‐PAS identified additional TTF‐1‐negative ADCs that would otherwise have remained difficult to subclassify. The authors also reported minor mucin positivity in a small subset of SqCCs [8]. In contrast, we observed no PAS‐D positivity in SqCC cases when strict interpretation criteria were applied. Based on our experience, cytoplasmic glycogenization, cytoplasmic condensation of dyskeratotic cells and entrapped benign glands may account for false‐positive mucin staining, underscoring the importance of careful morphology‐driven interpretation.

TTF‐1, the most effective pneumocyte marker associated with surfactant apoprotein, serves as a marker of terminal respiratory unit (TRU)‐derived tumors. Tumors arising from peripheral airways and small bronchioles are typically TTF‐1 positive because of their TRU origin [13, 14]. Accordingly, TTF‐1 remains indispensable for diagnosing ADC in both primary and metastatic settings. However, identifying ADCs that lack TTF‐1 expression remains a major diagnostic challenge. Napsin A offers additional value because both markers largely identify TRU‐derived tumors and numerous studies have reported near‐perfect concordance (99%–100%) between TTF‐1 and Napsin A expression [15, 16, 17]. Conversely, centrally located ADCs arising from non‐TRU origins are more likely to be TTF‐1 negative while retaining positivity for histochemical mucin stains [13, 14], explaining the diagnostic utility of PAS‐D in this setting.

Importantly, PAS‐D positivity should not be interpreted as evidence of primary pulmonary origin. Whereas TTF‐1 supports both pulmonary lineage and adenocarcinoma differentiation, PAS‐D primarily reflects intracellular mucin production and does not provide lineage information. Since mucin production is a common feature shared by adenocarcinomas arising from various organs, PAS‐D should be regarded as a marker of glandular differentiation rather than a marker of tumor origin. This distinction is particularly important in TTF‐1‐negative tumors, in which metastatic ADCs may closely mimic primary pulmonary ADC. Accordingly, PAS‐D findings should always be interpreted within an integrated clinicopathologic framework incorporating morphology, clinical history, radiologic findings, and lineage‐specific immunohistochemical markers.

These biologic concepts were further supported by our specimen‐based analysis. Among TTF‐1‐negative ADCs, PAS‐D maintained consistently high positivity across all specimen types, whereas Napsin A provided limited additional diagnostic information. These findings suggest that once TTF‐1 expression is lost, TRU‐associated markers lose much of their diagnostic utility, while PAS‐D largely preserves its ability to demonstrate glandular differentiation. Importantly, these observations do not challenge the central role of TTF‐1/p40‐based algorithms, but rather reinforce the complementary role of PAS‐D in bridging a persistent diagnostic gap that remains unresolved by lineage‐specific markers alone.

Because ADCs frequently arise in the peripheral lung parenchyma, cytology plays a critical role in diagnosis, particularly transthoracic FNAs and pleural effusions. Nevertheless, few studies have evaluated mucin stains in cytology‐based diagnostic algorithms. Most cytological studies rely exclusively on IHC alone, confirming Napsin A positivity in TTF‐1‐positive cases without benefit in TTF‐1‐negative ADCs [18, 19, 20, 21]. To our knowledge, Nicholson et al. conducted the only study incorporating PAS‐D mucin stain into cytologic NSCLC diagnosis and recommended combining TTF‐1 with PAS‐D to identify both central and peripheral ADCs [14]. Our findings support this approach, as PAS‐D proved particularly useful for identifying TTF‐1‐negative ADCs in cytologic specimens.

LCC remains a diagnosis of exclusion that can only be definitively established in resection specimens following extensive ancillary testing. Molecular studies have shown that many tumors previously classified as LCC exhibit ADC‐like molecular profiles, suggesting that a substantial proportion may represent incompletely characterized adenocarcinomas rather than a distinct biological entity [22, 23, 24, 25, 26]. Consistent with previous reports, our retrospective re‐evaluation revealed that a significant proportion of tumors initially classified as LCC were reclassifiable according to contemporary criteria, emphasizing the importance of standardized diagnostic approaches. In line with previous studies, many reclassified ADCs showed partial immunohistochemical staining patterns, highlighting the impact of intratumoral heterogeneity on definitive subclassification [21, 23]. These findings suggest that more extensive sampling and comprehensive ancillary evaluation across multiple tissue blocks may improve classification accuracy in poorly differentiated tumors. Furthermore, rhabdoid morphology should prompt consideration of recently recognized thoracic tumors involving SWI/SNF complex alterations, particularly SMARCA4‐ or SMARCB1 (INI1)‐deficient tumors, in the differential diagnosis [27, 28].

The present study has several notable strengths, including its large cohort size, evaluation by expert pulmonary pathologists/cytopathologists, and systematic verification of tumor origin through clinicopathologic and radiologic correlation, ensuring that all analyzes were performed on a well‐defined cohort of confirmed primary pulmonary tumors. The inclusion of all specimen types encountered in routine pulmonary pathology practice represents both a strength and a potential limitation of the study. Although specimen‐related differences may influence histologic interpretation and ancillary test performance, this approach enhances the real‐world relevance, clinical applicability, and generalizability of our findings. Nevertheless, several limitations should be acknowledged, including the retrospective design, the non‐uniform application of ancillary stains over time, and the limited availability of comprehensive molecular data.

Although PAS‐D is an established histochemical technique, its role in contemporary diagnostic practice warrants renewed attention. Our results indicate that underutilization of PAS‐D may inadvertently contribute to incomplete NSCLC subclassification, particularly in TTF‐1‐negative tumors and limited specimens. Rather than replacing established TTF‐1/p40 short panel algorithm, these results support optimizing current diagnostic workflows through the reintroduction of a simple, widely available ancillary method that remains underused in routine practice.

To our knowledge, this is the largest real‐world study to date demonstrating the diagnostic contribution of PAS‐D to NSCLC subclassification across cytology, small biopsy, and resection specimens over a 10‐year period. In conclusion, PAS‐D represents a practical complementary tool for NSCLC subclassification, particularly in diagnostically challenging TTF‐1‐negative ADCs. Its incorporation into contemporary diagnostic workflows may enhance subclassification accuracy while preserving tissue for downstream molecular analyzes, thereby supporting more confident clinical decision‐making in challenging cases.

## Author Contributions

DVB and YO designed the study. DVB, HO, and MB collected the data. DVB, DY, and YO analyzed the data. DVB and YO wrote the article. All authors reviewed, contributed and edited the final draft. All authors approved the final version.

## Funding

The authors have nothing to report.

## Ethics Statement

The study was approved by the Clinical Research Ethics Committee of Istanbul University, Istanbul Faculty of Medicine (2024‐176).

## Conflicts of Interest

The authors declare no conflicts of interest.

## Data Availability

The data that support the findings of this study are available from the corresponding author upon reasonable request. The authors declare that the data can be available upon the request of the journal.

## References

[1] G. V. Scagliotti , U. Pastorino , J. F. Vansteenkiste , et al., “A Phase III Randomized Study of Surgery Alone or Surgery Plus Preoperative Gemcitabine‐Cisplatin in Early‐Stage Non‐Small Cell Lung Cancer (NSCLC): Follow‐Up Data of Ch.E.S,” Journal of Clinical Oncology 26 (2008): 7508. 10.1200/JCO.2010.33.7089 22124104

[2] D. H. Johnson , L. Fehrenbacher , W. F. Novotny , et al., “Randomized Phase II Trial Comparing Bevacizumab Plus Carboplatin and Paclitaxel With Carboplatin and Paclitaxel Alone in Previously Untreated Locally Advanced or Metastatic Non‐Small‐Cell Lung Cancer,” Journal of Clinical Oncology 22 (2004): 2184–2191. 15169807 10.1200/JCO.2004.11.022

[3] WHO E ., WHO Classification Thoracic Tumours (IARC Press, 2021).

[4] J. A. Bishop , J. Teruya‐Feldstein , W. H. Westra , G. Pelosi , W. D. Travis , and N. Rekhtman , “p40 (ΔNp63) Is Superior to p63 for the Diagnosis of Pulmonary Squamous Cell Carcinoma,” Modern Pathology 25 (2012): 405–415. 22056955 10.1038/modpathol.2011.173

[5] N. Rekhtman , D. C. Ang , C. S. Sima , W. D. Travis , and A. L. Moreira , “Immunohistochemical Algorithm for Differentiation of Lung Adenocarcinoma and Squamous Cell Carcinoma Based on Large Series of Whole‐Tissue Sections With Validation in Small Specimens,” Modern Pathology 24 (2011): 1348–1359. 21623384 10.1038/modpathol.2011.92

[6] W. D. Travis , E. Brambilla , A. P. Burke , A. Marx , and A. G. Nicholson , “Introduction to the 2015 World Health Organization Classification of Tumors of the Lung, Pleura, Thymus, and Heart,” Journal of Thoracic Oncology 10 (2015): 1240–1242. 26291007 10.1097/JTO.0000000000000663

[7] W. D. Travis , E. Brambilla , M. Noguchi , et al., “International Association for the Study of Lung Cancer/American Thoracic Society/European Respiratory Society International Multidisciplinary Classification of Lung Adenocarcinoma,” Journal of Thoracic Oncology 6 (2011): 244–285. 21252716 10.1097/JTO.0b013e318206a221 PMC4513953

[8] P. Micke , J. Botling , J. S. M. Mattsson , et al., “Mucin Staining Is of Limited Value in Addition to Basic Immunohistochemical Analyses in the Diagnostics of Non‐Small Cell Lung Cancer,” Scientific Reports 9 (2019): 1319. 30718697 10.1038/s41598-018-37722-0 PMC6362145

[9] A. Kennedy and P. D. Burgin , “A Comparison of Different Methods of Detecting Mucin in Adenocarcinomas of the Lung,” British Journal of Diseases of the Chest 69 (1975): 137–143. 49191

[10] D. H. McGregor , A. Y. Dixon , and D. K. McGregor , “Adenocarcinoma of the Lung: A Comparative Diagnostic Study Using Light and Electron Microscopy,” Human Pathology 19 (1988): 910–913. 3402980 10.1016/s0046-8177(88)80005-4

[11] M. R. Wick , T. Loy , S. E. Mills , J. F. Legier , and J. C. Manivel , “Malignant Epithelioid Pleural Mesothelioma Versus Peripheral Pulmonary Adenocarcinoma: A Histochemical, Ultrastructural, and Immunohistologic Study of 103 Cases,” Human Pathology 21 (1990): 759–766. 2193875 10.1016/0046-8177(90)90036-5

[12] P. S. Loo , S. C. Thomas , M. C. Nicolson , M. N. Fyfe , and K. M. Kerr , “Subtyping of Undifferentiated Non‐Small Cell Carcinomas in Bronchial Biopsy Specimens,” Journal of Thoracic Oncology 5 (2010): 442–447. 20195168 10.1097/JTO.0b013e3181d40fac

[13] Y. Yatabe , T. Mitsudomi , and T. Takahashi , “TTF‐1 Expression in Pulmonary Adenocarcinomas,” American Journal of Surgical Pathology 26 (2002): 767–773. 12023581 10.1097/00000478-200206000-00010

[14] A. G. Nicholson , D. Gonzalez , P. Shah , et al., “Refining the Diagnosis and EGFR Status of Non‐Small Cell Lung Carcinoma in Biopsy and Cytologic Material, Using a Panel of Mucin Staining, TTF‐1, Cytokeratin 5/6, and P63, and EGFR Mutation Analysis,” Journal of Thoracic Oncology: Official Publication of the International Association for the Study of Lung Cancer 5 (2010): 436–441. 20068475 10.1097/JTO.0b013e3181c6ed9b

[15] B. M. Turner , P. T. Cagle , I. M. Sainz , J. Fukuoka , S. S. Shen , and J. Jagirdar , “Napsin A, a New Marker for Lung Adenocarcinoma, Is Complementary and More Sensitive and Specific Than Thyroid Transcription Factor 1 in the Differential Diagnosis of Primary Pulmonary Carcinoma: Evaluation of 1674 Cases by Tissue Microarray,” Archives of Pathology and Laboratory Medicine 136 (2012): 163–171. 22288963 10.5858/arpa.2011-0320-OA

[16] S. Mukhopadhyay and A.‐L. A. Katzenstein , “Subclassification of Non‐Small Cell Lung Carcinomas Lacking Morphologic Differentiation on Biopsy Specimens: Utility of an Immunohistochemical Panel Containing TTF‐1, Napsin A, p63, and CK5/6,” American Journal of Surgical Pathology 35 (2011): 15–25. 21164283 10.1097/PAS.0b013e3182036d05

[17] J. A. Bishop , H. Benjamin , H. Cholakh , A. Chajut , D. P. Clark , and W. H. Westra , “Accurate Classification of Non–Small Cell Lung Carcinoma Using a Novel MicroRNA‐Based Approach,” Clinical Cancer Research 16 (2010): 610–619. 20068099 10.1158/1078-0432.CCR-09-2638

[18] T. Sharma , P. Das , R. Panigrahi , C. M. Rao , and J. Rath , “Immunocytochemical Evaluation of TTF‐1, Napsin‐A, and p‐63 for Subtyping of Non‐Small Cell Lung Carcinoma and Clinicopathological Correlation,” Journal of Cytology 39 (2022): 180–187. 36605876 10.4103/joc.joc_5_22 PMC9809422

[19] A. van Zyl , P. T. Schubert , and C. F. N. Koegelenberg , “The Utility of TTF‐1, Napsin A, CK5 and p63 Staining in the Sub‐Classification of Non‐Small Cell Carcinoma of the Lung,” Cytopathology 30 (2019): 586–591. 31206846 10.1111/cyt.12741

[20] G. T. Gurda , L. Zhang , Y. Wang , et al., “Utility of Five Commonly Used Immunohistochemical Markers TTF‐1, Napsin A, CK7, CK5/6 and P63 in Primary and Metastatic Adenocarcinoma and Squamous Cell Carcinoma of the Lung: A Retrospective Study of 246 Fine Needle Aspiration Cases,” Clinical and Translational Medicine 4 (2015): 16. 25977750 10.1186/s40169-015-0057-2 PMC4417108

[21] L. Righi , P. Graziano , A. Fornari , et al., “Immunohistochemical Subtyping of Nonsmall Cell Lung Cancer Not Otherwise Specified in Fine‐Needle Aspiration Cytology: A Retrospective Study of 103 Cases With Surgical Correlation,” Cancer 117 (2011): 3416–3423. 21246522 10.1002/cncr.25830

[22] N. Rekhtman , L. J. Tafe , J. E. Chaft , et al., “Distinct Profile of Driver Mutations and Clinical Features in Immunomarker‐Defined Subsets of Pulmonary Large‐Cell Carcinoma,” Modern Pathology 26 (2013): 511–522. 23196793 10.1038/modpathol.2012.195 PMC3594043

[23] M. Barbareschi , C. Cantaloni , V. Del Vescovo , et al., “Heterogeneity of Large Cell Carcinoma of the Lung,” American Journal of Clinical Pathology 136 (2011): 773–782. 22031317 10.1309/AJCPYY79XAGRAYCJ

[24] D. H. Hwang , D. P. Szeto , A. S. Perry , J. L. Bruce , and L. M. Sholl , “Pulmonary Large Cell Carcinoma Lacking Squamous Differentiation Is Clinicopathologically Indistinguishable From Solid‐Subtype Adenocarcinoma,” Archives of Pathology and Laboratory Medicine 138 (2014): 626–635. 23738762 10.5858/arpa.2013-0179-OA

[25] G. Rossi , M. C. Mengoli , A. Cavazza , et al., “Large Cell Carcinoma of the Lung: Clinically Oriented Classification Integrating Immunohistochemistry and Molecular Biology,” Virchows Archiv 464 (2014): 61–68. 24221342 10.1007/s00428-013-1501-6

[26] J. Hou , M. Lambers , B. den Hamer , et al., “Expression Profiling‐Based Subtyping Identifies Novel Non‐Small Cell Lung Cancer Subgroups and Implicates Putative Resistance to Pemetrexed Therapy,” Journal of Thoracic Oncology 7 (2012): 105–114. 22134068 10.1097/JTO.0b013e3182352a45

[27] N. Rekhtman , J. Montecalvo , J. C. Chang , et al., “SMARCA4‐Deficient Thoracic Sarcomatoid Tumors Represent Primarily Smoking‐Related Undifferentiated Carcinomas Rather Than Primary Thoracic Sarcomas,” Journal of Thoracic Oncology 15 (2020): 231–247. 31751681 10.1016/j.jtho.2019.10.023 PMC7556987

[28] M. Haberecker , M. M. Bühler , A. P. Mendieta , et al., “Molecular and Immunophenotypic Characterization of SMARCB1 (INI1) ‐ Deficient Intrathoracic Neoplasms,” Modern Pathology 35 (2022): 1860–1869. 35864317 10.1038/s41379-022-01133-4 PMC9708576

